# Pathology and Practice *(Medical)*

**Published:** 1818-02

**Authors:** 


### II. PATHOLOGY and PRACTICE *(Medical).*

1. *Diseases of the respiratory Organs.* The distinction of active inflammation os the larynx from similar affections of the surrounding parts, constitutes an acquisition, of recent date and no mean importance, to our pathological knowledge. During the last two or three years, several cases of it have been recorded in the British Medical Journals, and other miscellaneous publications; ‡ and with the ardour and decision which characterize the professional men of this country, the only plan of treatment, apparently calculated to arrest the progress of this terrible disease, has been

---

‡ The more important communications on this subject, which we recollect to have seen in British works, are those of *Baillie* and *Farre*, *Medico-Chirurgical Transactions*, vol iii; *Percival, same work*, vol. iv; *Wilson, same work*, vol. v; *Armstrong, Edinburgh Med. and Surg. Journal*, vol. x; *Anon same work*, vol. xi; *Roberts, Med.-Chir. Trans.* vol. vi; *Fraser, London Medical Repository*, vol. v; *Robarts, same work*, vol. vi; *Abercrombie, Edin. Med. and Surg. Journal*, vol. xii; and *Johnson, Medico-Chirurgical Journal*, vol. v. See also valuable Observations on Laryngeal Diseases, by *Lawrence, Med.-Chirurg. Trans.* vol. vi; *Bedingfield, Compendium of Medical Practice*, chap. 6, and *Lond. Med. Repository*, vol. v.

promptly instituted, and, in a majority of instances, successfully pursued. Taking a retrospect of the medical literature of the Continent during the period just alluded to, we are struck with the silence, which there prevails upon this subject, compared with the lively interest and attention excited by it here. Indeed, the ensuing* is the only original and circumstantially detailed case which we recollect to have seen in the numerous foreign Journals, passing monthly under our review. On the inert and inefficient practice which it exhibits, comment would be superfluous. In February, 1817, a soldier, aged 29, was seized, during convalescence from fever apparently of typhoid character, with hoarseness and occasional cough. Expectoration of viscid mucus, mixed with nearly limpid saliva, was effected without effort. He ate well, walked out, and displayed no other sign of indisposition. Some trifling "pectoral" remedies were prescribed. On the evening of the 18th, the hoarseness was aggravated; inspiration very difficult; and, at times, a noise heard resembling that which accompanies a violent asthmatic paroxysm. The patient then first complained of a sense of obstruction about the larynx, occasionally preventing the passage of the air. The pulse continued natural. Reflection upon these symptoms at length conducted the physician, Dr. Ducasse, to a correct diagnosis of the malady. Aqueous solutions of tartrite of antimony, *antispasmodics* and *wine*, were the remedies prescribed. The patient continually called out for food, and evinced no uneasiness respecting his situation. On the 25th, in consequence of a severe suffocative paroxysm, which had nearly proved fatal, leeches were applied to the lateral regions of the throat, and, soon afterwards, a sinapism to the anterior. An addition of sulphuret of potash was also made to a "pectoral potion" previously employed. Slight amelioration of the symptoms was speedily followed by a fresh paroxysm of suffocation, and aphonia. Henceforth, respiration was constantly difficult; and a noise, extremely painful to the by-standers, accompanied inspiration. It was now thought proper to provide a canula for introduction into the glottis in the event of an emergency. But the disease made such accelerated progress, and "the last paroxysms had so fatigued the lungs and exhausted the patient," that the case was abandoned as desperate, without any attempt at relief by the introduction of the canula, or the operation of laryngotomy. Death took place in the interval of a paroxysm, on the 6th of March.

*Dissection.* The heat of the body, and flexibility of the limbs,

---

* *Observation d'une angine laryngée œdémateuse; par M. Ducasse,* &c. *Journal universel des sciences médicales,* tome v. A very concise notice of this malady is delivered in the second volume of the *Dictionnaire des Sciences Médicales,* under the article *Angine;* and an elaborate history of it by Bayle, in the 18th vol. of the same work. This we shortly propose to analyse. An Essay, published by Thuillier, *Sur l'angine laryngée œdémateuse,* we have not yet seen. Editors.

continued only a few hours after death.* The pharynx was perfectly sound; but such was the tumefaction of the whole circumference of the glottis, that the little finger could not be introduced into the cavity of the larynx. From incisions of the tumefied parts, made in various directions, oozed out a whitish whey-like fluid. The internal membrane of the larynx appeared slightly swollen, and its blood-vessels much injected. In traversing this membrane with the scalpel point, there suddenly burst out from a small incision on the right of the larynx, a considerable quantity of pus, the focus of which occupied a large portion of this side of the organ. The posterior border of the thyroid cartilage, and part of the arytenoid of that side, exhibited several black spots. Examination was carried no farther. We find nothing in the "*Reflections*" of the author or editor, upon this case, worthy of transcription.

To the moxa, as a remedy little known or prescribed in this country, we have repeatedly endeavoured to direct the attention of our readers. Instances of its successful operation in articular and neuralgic diseases, and in various formidable affections of the internal organs, have been successively quoted by us, upon the authority of reputable foreign writers.† We trust that our efforts to accelerate the introduction of a novel and important medicinal agent into British practice, will not prove utterly abortive. Much has recently been said, in the continental Journals, on the successful treatment of *pulmonary consumption* by moxa: and, although none can view with an eye of deeper scepticism and hopelessness than ourselves, newly proposed remedies for this deplorable malady, yet, from the full conviction that the institution of extensive purulent drains on the parietes of the thorax forms one of the most effectual means whereby its progress may be retarded, we really think that the moxa is entitled to a distinction above the common crowd of antidotes and specifics yearly obtruded on the public notice; and that it should, at least, previously to rejection, be submitted to the ordeal of fair experiment.‡ The following instance of its efficacy in "confirmed pulmonary phthisis"§ is subjoined, as one of the most striking and decisive which we have yet found upon record. A youth, aged 18, employed in the coral fishery, became affected with severe and deep-seated pains about the centre of the sternum, and between the shoulders. Loss of appetite, ex-

---

* Dr. Ducasse supposes that the subjects, upon whom the contrary of these two circumstances has been remarked by Bayle, had died in the first paroxysms of suffocation.

† See *Medico-Chir. Journal and Review*, vol. iv. page 334.

‡ At all events, the moxa will, in general, we are persuaded, be found preferable to the knife, the caustic, or the blistering fly, in the formation of a purulent drain. Edit.

§ *Observation sur une phthisie pulmonaire confirmée, guérie par le moxa*, &c. *par J. A. Saissy, D. M.* &c. *Recueil périodique de la Société de Médecine de Paris*, Aout 1817.

treme weakness, emaciation, flushing of the cheeks, frequent and obstinate cough with *purulent* expectoration, sense of suffocation on the least accelerated motion, hectic fever with evening exacerbations, profuse night sweats, and curvature of the nails, were the other principal symptoms. They had succeeded the disappearance of itch, and hence were regarded as the consequence of the repelled eruption. Blisters, and many other remedies, had been unavailingly prescribed. After fruitless attempts to reproduce the cutaneous affection by inoculation, and contact with the linen and persons of infected subjects, a large moxa was burnt on the centre of the sternum. In an hour after its employment, the patient experienced a fever fit, of ten hours' duration; and, meanwhile, perspired and expectorated copiously. A profound sleep, of six hours, succeeded the paroxysm; and the patient found himself, on awaking, unusually refreshed. Henceforth the fever declined: the expectoration lost its purulent character in eight days from the application of the moxa, although the discharge of mucus continued abundant a week longer. In fine, all the symptoms rapidly disappeared, and the health and vigour of the young man were perfectly re-established. The detachment of the eschar, on the fifteenth day, was followed by profuse suppuration. The pus was so "acrimonious as to corrode the borders of the ulcer, and excite an erysipelatous eruption, and pustules resembling those of itch, on the skin with which it came in contact. The sore assumed the form and character of psoric ulcers." Six weeks after the separation of the eschar, a small portion of the sternum exfoliated.

In three weeks, the young man left the hospital, and was so far recovered as to accompany a fishing party to the Moorish coast; from whence, in consequence of being fired at by the natives, he was under the necessity of swimming nearly the eighth of a league to his boat. His recovery was permanent; and no internal remedy appears to have been employed, to which this fortunate result can, in the slightest degree, be attributed. It is evidently the opinion of Dr. Saissy, the narrator of this case, that the moxa exercises a general, as well as topical operation. "Its action (he observes) is slow and gradual. The caloric extricated from it, penetrates deeply into the structure of the corresponding parts. Perhaps, even there is no point of the whole system to which the fire does not penetrate by irradiation. Of this, the fever, excited some minutes after the application of the moxa, constitutes a proof."

2. *Abdominal Diseases.* The important "Cases in elucidation of the Theory of Tympanitis,* cursorily adverted to in our last month's Sketch, we now proceed to analyze. They are three in number. It were almost needless to observe what a new and interesting field

* *Observations propres à eclairer la théorie de la Tympanite; par M. Bricheteau, D.M. Bulletin de l'Athénée de Médecine de Paris,* Novembre 1816.

in the pathology of intestinal affections, such facts, contemplated in their proper light, disclose; or how far they add weight and confirmation to the simple and correct doctrine, daily gaining ground among us, that all the countless varieties of chronic, nervous, convulsive, and spasmodic disease, are essentially connected, in their origin, with increased vascular action.*

About two years ago, a man, affected with tympanitis, was admitted into the *Hôtel-Dieu*, at Paris. Different modes of treatment were employed without success; nor could the cause of the intestinal dilatation be ascertained. At length the man died, and, on dissection, was discovered a contraction of the inferior part of the left iliac (descending) colon, consequent on chronic inflammation, and almost completely obstructing the canal of the intestine.

---

* The writer of the Sketch has long been turning the closest attention, of which his mind is capable, to that class of intestinal affections in which the morbid generation of flatus constitutes one of the more prominent phænomena; convinced that, to the distension of the intestinal canal by the gas extricated therein, or to the state of bowels connected with such extrication, many distressing and otherwise inexplicable symptoms, of a nervous, hysterical, and hypochondriacal description, may be correctly referred; and are only to be permanently remedied by treatment particularly directed to this source. In numerous instances, he has been enabled, by a rigorous system of moral and dietetic restriction, and the employment of alkaline and purgative medicines, to obviate the generation of flatulence, and subvert the train of morbid sensations intimately, and he thinks essentially, connected with it. Several cases, however, have recently occurred, in which this treatment has been long and steadily followed without success. The attention of the writer thus more strongly than ever excited to the subject, he has, at length, found, that *flatulent affections have commonly for their source, or are closely allied with, congestion or chronic inflammation of the mucous membrane of the intestinal canal, and more particularly that of the transverse and descending colon.* And he is, farther, experimentally convinced, that, whenever such affections resist the properly continued operation of the remedies above specified, *local blood-letting* and *counter-irritants* may be most advantageously employed; and will, in fact, remove them, if chronic thickening of the intestinal parietes have not taken place, or disorganization be not absolutely impending. How far the facts, detailed by Dr. Bricheteau, go in support of these conclusions, it were useless for the writer to point out: nor can any one, less deeply devoted than himself to the study of medicine, conceive the interest and avidity with which he perused the paper wherein they are consigned. The salutary revolution which such views and observations are calculated to effect in the treatment of colic and flatulent complaints, is sufficiently obvious. Abstinence and purgatives, the lytta and the leech, will, it is sincerely to be hoped, be, ere long, generally substituted for the opiates, æthers, and stimulants, and all the precious carminative and antispasmodic formulæ of old-school practice; which, while they afford delusive relief, serve only to augment the obstinacy of the disease, and accelerate its tardy but destructive progress. Upon this subject, some tolerably conclusive evidence will shortly be laid before the medical public, whose attention is meanwhile most earnetsly solicited to it,—by one of the Editors.

A woman, aged 70, who had previously enjoyed good health, and never suffered from severe disease, became subject to frequent attacks of colic, which were relieved by injections. On the 10th of March, six months from the first seizure, the disease became more violent, and no longer allowed of mitigation. After a full meal on the 13th, the woman was attacked with copious vomiting of food and mucus, which continued the whole night. The abdomen also became painful, and began to swell. On the 18th, the period of her admission into the hospital, the patient's abdomen was voluminous, tense, and sounded, on percussion, like a drum; but it was not painful to the touch. The patient, however, complained, at times, of colic. Her countenance was somewhat shrunk; her pulse natural. The non-existence of fœcal accumulation in the rectum was ascertained by introduction of the finger. Diluents, laxatives, and application of leeches to the anus, were now prescribed with relief to the patient; who said that she had been at stool, and discharged much flatus both by the rectum and œsophagus. On the fourth day of her admission, vomiting of greenish fluid supervened; and the same treatment was continued. On the 5th, the vomiting was unabated, the face constantly shrunk, and the abdomen very large. Frictions with camphorated spirit directed. On the 6th and 7th, the abdomen was farther increased in size, and painful on pressure. Sleeplessness, vomiting, constipation. Leeches were applied to the anus, warm fomentations to the abdomen, and injections. All the symptoms were, on the 8th, aggravated. Death took place on the 9th.

*Dissection.* The abdomen, very tense and voluminous, sounded like a drum. Its parietes, although belonging to a corpulent subject, were, from distension, reduced to one-third of an inch in thickness. The intestines, with the exception of the rectum, were slightly inflamed and inordinately dilated. Much fœtid gas, and yellowish, liquid fæcal matter, escaped from them. The rectum, collapsed, exhibited a comparative state of contraction. On examination of the inferior part of the sigmoid flexure of the colon, where the intestinal distension terminated, an annular contraction was seen, formed by the thickened mucous membrane, and scarcely allowing the introduction of the little finger. It exhibited, upon incision, no appearance of carcinomatous structure. Dr. Bricheteau thinks it probable that this contraction had interrupted the continuity of the intestinal tube, and that much gas had been extricated from the accumulation of fæces.

A very corpulent female, aged forty, of good constitution and lymphatic temperament, was admitted into the *Hotel-Dieu*, July 29th, 1816. She complained of wandering pains in the abdomen, which was somewhat painful on pressure, and had acquired an unusual volume. To these symptoms were added a pale and bloated countenance. The condition of the patient was ameliorated by the general employment of emollients and sedatives, but the abdomen remained constantly swollen. Diarrhœa and constipation alternated with each other. The case otherwise exhibited no alarming feature. Sometimes it was stationary, and scarcely attracted the physician's notice. Towards the close of July, however, the intestinal pains reappeared with much violence, and the abdomen became more swollen. The patient suddenly expired in a state of exhaustion, on the 1st of September.—*Dissection.* The colon, cæcum, and whole track of the small intestine, were so much distended that the abdominal parietes could not be incised without wounding them. Yet they, like all the viscera of the abdomen, displayed, at first sight, a healthy appearance. Much gas escaped from the intestines; the distension of which terminated at the inferior part of the descending colon: and there the intestine had contracted adhesions with the summit of the iliac fossa. Slight force, employed in the examination of the adherent gut, sufficed to rupture it; and a black offensive fluid issued from the opening. It was then seen that the colic flexure had formed, in consequence of the adhesion, a *cul-de-sac*, in which fæcal matter had accumulated. Below, was developed in the intestinal mucous membrane, an organic lesion, which seemed closely to resemble the scirrhous state induced by chronic inflammation; and greatly contracted the canal of the intestine. From the rupture of the gut, it was impossible to ascertain the state of the morbid portion communicating with the rectum; and whether the continuity of the canal had been wholly interrupted.

In the three preceding cases, observes Dr. Bricheteau, the tympanitis was symptomatic of *chronic inflammation of the colon;* and he considers it remarkable that, in all of them, the fatal lesion was found at the same point of the intestinal canal.* From the premises just stated, he is also led to infer that intestinal tympanitis, of more common occurrence than is generally supposed, most frequently originates from organic lesions obstructing, or obliterating the alimentary canal; and that the gas which distends the intestines, is disengaged from the fæcal matter accumulated in them.

---

* This circumstance will no longer appear singular, when we reflect that fæcal accumulation, if it exist at all, invariably commences in, and is commonly confined to, the flexure and descending portion of the colon. Constipation is by far the most frequent cause of this class of diseases: and hence it is that women, in the middle and higher classes, upon whom the present state of society imposes so many unnatural and injurious restrictions, constitute a great majority of its victims. A collection of fæcal matter takes place in, and irritates, the descending colon. The state of distension, thus induced, is followed by a congestive, or chronically inflamed condition of the mucous membrane; and farther aggravated by the consequent extrication of gas and attendant spasm. And this, according to the temperament and habits of the patient, and the inert or stimulating treatment pursued, at length, terminates in incurable contraction, or fatal disorganization of the intestine. Colic diseases, we are in the habit of constantly seeing mistaken for affections of the liver, stomach, spleen, or kidney: and, of course, mal-treated.—Edit.

Dr. Bricheteau's concluding paragraph contains an intimation upon which the retrospect of many a painful event in our own practice, induces us to stamp no ordinary value.

"These facts, moreover, prove how difficult the diagnosis, how obscure the nature, and frequently fatal the termination of intestinal diseases. With many others, they conspire to evince the excellence of the advice of the celebrated Albertinus, who cautioned his pupil, Morgagni, to be constantly on his guard against intestinal pains; affirming, that he had sometimes seen, after *slight pains, unaccompanied by evident pyrexia or vomiting*, death suddenly ensue from *latent inflammation of the bowels.*"

Ere we quit this section, it may not be improper to take brief notice of a case of abdominal inflammation terminating in two considerable collections of matter, which spontaneously discharged the one by the rectum, the other by the urinary passages.* The subject of this observation was suddenly seized with violent pain in the right side of the abdomen, shooting towards the epigastrium, urgent thirst and vomiting, and incessant agitation. The pulse was hard and febrile; the countenance dejected; the abdomen contracted and intolerant of the slightest pressure. The diluents, prescribed on the suspicion of intestinal inflammation, were rejected without mixture. The introduction of glysters was prevented by the abdominal constriction, and warm fomentations aggravated the pain; which, however, was relieved by two general bleedings, and the employment of the tepid bath. Glysters and fomentations were resumed with better effect. On the fourteenth day, there were irregular shiverings with sleeplessness. The tongue was coated with a whitish fur; the skin dry; the urine copious and red. A large sinapism was now applied to the bowels. The pain, following the course of the transverse and descending colon, fixed in the left hypochondrium. The thirst and fever now subsided, and sleep was restored, although the abdominal tension continued. The fæces constantly exhibited a covering of mucus, and the urine could only be voided in certain positions of the body. The patient, when standing erect, experienced a sense of weight at the anus, with inclination to go to stool. All these symptoms were regarded as announcing the formation of an abscess in the duplicature of the mesentery near the bladder. Soon afterwards, a tumour arose in the left iliac region, extending towards the hypogastrium, and gradually enlarging. By the introduction of a finger into the rectum, pressure meanwhile being made with the other hand on the abdomen, a round and resistent tumour was discovered. Shiverings experienced towards night, and a brown state of the tongue, confirmed the diagnosis. At length, upon an effort to void fæces, about one pint of white and inodorous pus escaped from the anus.

---

* *Observation sur une Inflammation du bas-ventre*, &c. Par M. Fauverge, Medecin à Mayence.—*Recueil period. de la Société de Méd. de Paris*, Aout 1817.

The patient now seemed quite well; and, in one week, the purulent discharge from the rectum subsided. Shortly, however, a fit of anger was followed by fresh abdominal pains and occasional shiverings, which yielded to glysters and embrocations. But, soon afterwards, the lady was obliged, by a sense of weight in the abdomen, to suspend her walk. The pain and shivering returned; the part became sore; the stools scanty and difficult. The ordinary treatment in internal suppuration was continued two months, when an abscess presented beneath the abdominal integuments in the space between the umbilicus and right anterior iliac spine. Every night a febrile paroxysm, of two hours' duration, came on precisely at ten o'clock. In about twenty days, fluctuation was perceptible in the tumour; and, under the gentle pressure of the surgeon's finger, who was preparing to evacuate it, the sac burst internally, and poured its contents into the abdominal cavity. This event, in the exhausted state of the patient, was considered as almost inevitably fatal. Cinchona, wine, strong broths and jellies, were prescribed. Towards evening, fever with anxiety: disturbed night. On the morrow, pulse high; countenance livid and dejected; weakness extreme, but the night more calm. On the 3d, nature rose triumphant from the struggle. Much pus was deposited by the turbid urine. Amendment very perceptible; no fever. At the end of a week, the patient had lost all her complaints, except a pain felt daily for some hours, in the hypogastric region. This was attributed to the passage of excrement in the vicinity of the late seat of the abscess, or to adhesions consequent on it. A bland regimen, semicupia, and chamomile injections terminated the cure.

Pathology and Practice *(Surgical)*.—1. *Wounds.* The following "notice of a wound of the head, accompanied by singular phænomena,"* has recently been published by M. Larrey.—A man, aged 26, was struck, in fencing, with a foil, about the centre of the left canine region, near the ala nasi, in an oblique direction from below upwards, and rather from within outwards. The instrument penetrated to the depth of three inches and a half across the left nasal fossa, traversed the cribriform plate of the ethmoid bone near the insertion of the falx cerebri, and seemed to have entered in a vertical and somewhat oblique direction, to the depth of about six lines, into the internal posterior part of the left anterior cerebral lobe, very near the anterior of the middle lobe.—Copious hæmorrhage was an immediate consequence of the wound, and numerous fragments of bone issued from the mouth and nostrils. All the organs of sense were, at the moment, paralysed; but they have since gradually recovered their functions, and the following alterations only remain. The vision of the left eye, completely lost for

---

* *Note sur une plaie de tête, accompagnée de phénomenès singuliers;* par M. Le Baron Larrey. *Bulletin de la Faculté de Méd. de Paris,* 1817. No. vii.

a month, is now re-established; but all objects appear double. The smell is restored from total extinction, and the patient can now distinguish alcoholic from inodorous liquids. The taste, equally destroyed, has been gradually re-established on the right side of the tongue, while the left portion remains perfectly destitute of this faculty. The whole of the organ is drawn towards the right, in consequence of the hemiplegia of the right side, the mouth being distorted to the left. The function of the left ear, at first lost, has since been restored; and a buzzing noise only remains. The voice, which was also extinct, is recovered, with the exception of slight stammering. The generative organs retain all their vigour. There was, at first, hemiplegia of the whole right side; but paralysis of the right thoracic and abdominal extremities, as regards their locomotive faculty, now only remains: their sensibility is not impaired. The recollection of names was at first extinct, and is now but with difficulty recalled; while that of objects, and every thing susceptible of demonstration is in perfect integrity. The mental aberration, which, at first existed, has now ceased; but by all that relates to his vanity and military successes, he is plunged into a state of mental alienation and profound melancholy: while conversations, which have reference to his family and friends, restore to him his faculties. The patient recollects distinctly the person, figure, and features of M. Larrey. He should recognize him without difficulty: he is ever before his eyes (these are the patient's expressions). Yet he remembers not Larrey's name; and designates him by that of M. *Chose* (Mr. *Thingambey).*

An instance of *gun-shot wound*, followed, after a lapse of six weeks, by general emphysema,* will, doubtless, be read with interest. It conveys, as the author judiciously observes, an important lesson with respect to the care which latent and chronic inflammations in the vicinity of the lungs require; and proves the necessity of great circumspection in the prognosis delivered upon wounds of such parts, even when no menacing symptoms have, in the first instance, been developed.

In the last campaign of Italy, a German soldier received a musket-shot on the head of the left humerus; and, ten days afterwards, entered the hospital at Milan. The wound was then nearly cicatrized; the bone luxated and motionless, and the limb wasted. The ball had not been extracted. Neither fever, cough, hæmoptysis, dyspnœa, nor local pain existing; nothing was attempted. The wound completely closed, and the man was about to quit the hospital in a state of convalescence; when, on the 4th of June, he felt pain in the left pectoral muscle. An elevation, attributed to the presence of the ball, was there remarked, and the impending suppuration encouraged by poultices. Next day, the left shoulder displayed the same phænomenon; supposed also to be the

---

* *Emphyséme général, survenu un mois et demi après un coup de feu. Recueil period. de la Soc. de Méd. de Paris.* Avril 1817.

precursor of suppuration, although the pulse was natural. In the afternoon, the tumefaction extended to the whole shoulder and arm. On the 6th, it was opened; but, instead of pus, such as the first tumour had contained, bubbles of air only issued from the orifice. Saturnine applications were prescribed, and the tumour now recognized as emphysematous, was incised in various directions. On the morrow, the emphysema occupied the whole body to the fingers and toes. The eyes projected like those of a frog; and, in passing a finger over the radial artery, bubbles of air, resembling small ganglia, were felt. Transient relief was procured by extrication of air from incisions on the back and thorax. But the parts soon tumefied afresh, in an increased degree : and, after a struggle of three days, the patient sank.—*Dissection* was commenced at the head of the dislocated humerus, which was found split. On detaching the pectoralis major from the ribs, the fifth was seen fractured. When this was raised, the adherent pleura, and a superficial abscess in the part of the lung corresponding to the fracture, came into view. The ball not having been discovered beneath the pectoral muscles, nor in the lungs, or pleura, minute search was made; and it was, at last, found imbedded in the body of the fifth cervical vertebra. No internal extravasation or other lesion existed. The suppuration of the lung had probably been produced by the chronic inflammation, consequent on the violence which the ball had inflicted in its passage.

Pathology and Practice *(Obstetrical)*.—1. *Extra-Uterine Pregnancy.* The ensuing example of "parturition* per anum," lately observed by Professor Santorini, will require no introductory comment.—A healthy female, aged 30, became, for the fourth time, pregnant in the month of December. This state was attended with no particular symptom, except transient dysury. In March she sustained an attack of fever, with pain in the back, especially on walking; and the abdomen was so unusually large as to suggest the idea of the existence of twins. The lumbar pains were much aggravated by the motions of the fœtus. One night in May, the patient was seized with violent pains shooting from the abdomen to the loins, and menacing abortion. From the period of their cessation, the motions of the fœtus were no longer felt, and the abdomen diminished in volume. Obstinate constipation and convulsions resembling epilepsy, ensued. Vomiting was added to the fever, and the patient kept her bed. In July, the pains returned with such force that the midwife was summoned; but the figure of the abdomen, and state of the vagina, were not found to indicate labour. A trifling bloody discharge from the uterus succeeded, and afterwards became white and offensive. The pains again disappeared, but the discharge continued, at times, till Sep-

* *Accouchement par l'anus;* par le Professeur Santorini. *Recueil period. de la Soc. de Méd. de Paris.* Aout 1817.

tember. The mammæ were full of milk. From the head-ach, fever, nausea, watchfulness and constipation, extreme debility and frightful emaciation resulted. On examination, a small firm substance was felt in the left groin; but whether it were a fœtus or morbid tumour, could not be ascertained. All these symptoms, however, except the constipation and slight fever, ceased in September. The patient's strength returned, and the abdominal pain was only felt on strong pressure of the tumour. In January, menstruation was restored, and continued regular till July; when signs of fresh pregnancy re-appeared. The abdomen enlarged, and constipation ceased. Pain, as though a wedge were compressed between the sacrum and pubis, sometimes came on at night. About the middle of the following January, the patient was delivered of a seven months' child. The secretion of milk, and lochial flux, continued but eight days. The abdominal tumour, with pain on pressure, again shewed itself. In the commencement of March, having recently sustained a shock from the fall of her vehicle, the Lady arrived at Venice, with fever, head-ache, nausea, vomiting, diarrhœa, and a fixed pain in the sacrum. Violent efforts to vomit were followed by ejection of an ash-coloured and offensive fluid, and pungent pains, like those of a hard body penetrating the sacrum, were felt at the anus, on going to stool. The state of the uterus, upon examination, was natural. But the surgeon, introducing his finger into the rectum, discovered there a hard and sharp substance; and extracted with his forceps all the superior cranial bones of a fœtus, in succession. From the febrile and exhausted state of the patient, it was deemed proper to postpone, for a while, the completion of the process. At length, on the 20th of April, the remnant of a female fœtus was safely extracted. The body was, posteriorly, in a putrid condition; the occipital bone separated, and filled with indurated fæces. About six inches of funis adhered to the umbilicus. Henceforth, the abdominal tumour nearly subsided; but fever, thirst, nausea, frequent syncope, cough, and involuntary fæcal discharges supervened. On the morrow, a large mass of indurated excrement came away, and the tumour wholly disappeared. Much relief was derived from emollient glysters. A small placenta, with the yet attached relic of the funis passed by stool on the 27th. The cough, and all the other symptoms, with a subsequent erysipelas of the left cheek, gradually gave way; and, in six weeks, the Lady was convalescent.

The author of this case cursorily alludes to one more singular, the particulars of which it is his intention shortly to communicate. He has known both the *conception* and *extraction* of a fœtus take place *per anum;* the vagina opening into the rectum about one inch from the sphincter ani. The vulva here was imperforate, and formed only a fold of skin, two inches and a half long, and six lines deep."

